# Assessment of a Crowdsourcing Open Call for Approaches to University Community Engagement and Strategic Planning During COVID-19

**DOI:** 10.1001/jamanetworkopen.2021.10090

**Published:** 2021-05-14

**Authors:** Suzanne Day, Chunyan Li, Takhona Grace Hlatshwako, Fouad Abu-Hijleh, Larry Han, Chelsea Deitelzweig, Barry Bayus, Rohit Ramaswamy, Weiming Tang, Joseph D. Tucker

**Affiliations:** 1 Institute for Global Health and Infectious Diseases, University of North Carolina at Chapel Hill, Chapel Hill; 2 Department of Health Behavior, Gillings School of Global Public Health, University of North Carolina at Chapel Hill, Chapel Hill; 3 Department of Health Policy and Management, Gillings School of Global Public Health, University of North Carolina at Chapel Hill, Chapel Hill; 4 Department of Global Health, Gillings School of Global Public Health, University of North Carolina at Chapel Hill, Chapel Hill; 5 Department of Biostatistics, Harvard T.H. Chan School of Public Health, Boston, Massachusetts; 6 Department of English and Comparative Literature, University of North Carolina at Chapel Hill, Chapel Hill; 7 Kenan-Flagler Business School, University of North Carolina at Chapel Hill, Chapel Hill; 8 Public Health Leadership Program, Gillings School of Global Public Health, University of North Carolina at Chapel Hill, Chapel Hill; 9 Social Entrepreneurship to Spur Health, Guangzhou, China; 10 University of North Carolina at Chapel Hill Project–China, Guangzhou; 11 Dermatology Hospital, Southern Medical University, Guangzhou, China; 12 Department of Medicine, University of North Carolina at Chapel Hill, Chapel Hill; 13 Faculty of Infectious and Tropical Diseases, London School of Hygiene and Tropical Medicine, London, United Kingdom

## Abstract

**Importance:**

Reimagining university life during COVID-19 requires substantial innovation and meaningful community input. One method for obtaining community input is crowdsourcing, which involves having a group of individuals work to solve a problem and then publicly share solutions.

**Objective:**

To evaluate a crowdsourcing open call as an approach to COVID-19 university community engagement and strategic planning.

**Design, Setting, and Participants:**

This qualitative study assessed a crowdsourcing open call offered from June 16 to July 16, 2020, that sought ideas to inform safety in the fall 2020 semester at the University of North Carolina at Chapel Hill (UNC). Digital methods (email and social media) were used for promotion, and submissions were collected online for 4 weeks. Participation was open to UNC students, staff, faculty, and others.

**Main Outcomes and Measures:**

Submissions were evaluated for innovation, feasibility, inclusivity, and potential to improve safety and well-being. Demographic data were collected from submitting individuals, and submissions were qualitatively analyzed for emergent themes on challenges with and solutions for addressing safety and well-being in the fall semester. Data were shared with UNC leadership to inform decision-making.

**Results:**

The open call received 82 submissions from 110 participants, including current UNC students (56 submissions [68%]), people younger than 30 years (67 [82%]), women (55 [67%]), and individuals identifying as a racial/ethnic minority or as multiracial/ethnic (49 [60%]). Seven submissions were identified as finalists and received cash prizes with the encouragement to use these funds toward idea development and implementation. Seventeen runner-up teams were linked to university resources for further development. Thematic analysis of submissions regarding challenges with the fall semester revealed not only physical health concerns and the limitations of remote learning but also challenges that have been exacerbated by the pandemic, such as a lack of mental health support, structural racism and inequality, and insufficient public transportation. Solutions included novel ideas to support mental health among specific populations (eg, graduate students and racial/ethnic minorities), improve health equity, and increase transit access. All 24 finalists and runners-up indicated interest in implementation after being notified of the open call results.

**Conclusions and Relevance:**

This study suggests that open calls are a feasible strategy for university community engagement on COVID-19, providing a stakeholder-driven approach to identifying promising ideas for enhancing safety and well-being. Open calls could be formally incorporated into university planning processes to develop COVID-19 safety strategies that are responsive to diverse community members’ concerns.

## Key Points

**Question:**

Is a crowdsourcing open call a feasible approach to engaging the university community in COVID-19 safety strategies?

**Findings:**

This qualitative study evaluated 82 submissions to a university open call for creative solutions from students, faculty, and staff to inform safety in the fall 2020 semester. Solutions were shared with university leadership, and several are being further developed.

**Meaning:**

The results of this study suggest that open calls are a promising approach to understanding university community members’ concerns and identifying stakeholder-driven, innovative solutions for safe university activity during the pandemic.

## Teaser

This qualitative study evaluates a crowdsourcing open call as an approach to university community engagement and strategic planning to ensure safety during the COVID-19 pandemic.

## Introduction

The global COVID-19 pandemic has upended university life.^1^ Indoor, group-based, and in-person interactions are mainstays of the university experience but also facilitate COVID-19 transmission.^2^ Universities must resolve this tension to safely continue campus operations while addressing the concerns of students, staff, and faculty. In addition, COVID-19 has also profoundly impacted the methods used to develop new plans and engage the community.^3^ Furthermore, conventional university planning is often a top-down, expert-driven process with few opportunities for input from university community stakeholders.^4^

One promising community engagement method during COVID-19 has been to use a crowdsourcing open call. An open call provides a structured mechanism to aggregate wisdom from groups in response to a specific problem, and exceptional contributed solutions are then shared with the public.^5^ The US Office of Science and Technology Policy identified open calls as a centerpiece of the US Strategy for American Innovation, and the America COMPETES Reauthorization Act gives all government agencies the broad authority to conduct open challenges to promote innovation.^6^ Open calls have been used extensively by health and scientific research organizations as an innovative approach to problem solving, including the National Academies of Sciences, Engineering, and Medicine^7^ and the National Institutes of Health Office of Behavioral and Social Sciences Research.^8^ Randomized clinical trials have demonstrated the effectiveness of crowdsourcing across an array of health topics,^9^ and social science research has shown that open calls can enhance community engagement through the meaningful participation of a broad range of local stakeholders.^10,11^ For example, a crowdsourcing open call on the topic of HIV cure research resulted in creative contributions from local stakeholders and the development of messaging around HIV cure research that was both culturally appropriate and resonated with communities that are disproportionately impacted by HIV.^12^ In addition, compared with other community engagement mechanisms (eg, community advisory boards), crowdsourcing open calls have been found to engage a broader range of stakeholders whose perspectives are often underrepresented in health and medical research, including individuals with lower levels of education and lower incomes.^13^

Although there have been several crowdsourcing open calls to inform universities’ planning during COVID-19,^14,15,16,17^ none have been formally evaluated in terms of their process and outcomes. In addition, the limited existing research evaluating crowdsourcing open calls has not focused on emergency response to pandemics.^9^ This study seeks to address both of these gaps by presenting the results of a digital crowdsourcing open call to inform reopening processes at the University of North Carolina at Chapel Hill (UNC) during the COVID-19 pandemic. The purpose of this open call was to identify creative ideas for a safe fall semester through a community-engaged process. This article describes the design of this open call and analyzes the themes that emerged from participants’ submissions.

## Methods

### Open Call Design and Implementation

In the summer of 2020, our study team worked with a diverse group of UNC students, faculty, staff, and alumni to organize a crowdsourcing open call to inform increased safety in the fall semester during COVID-19. Operating as the Carolina Collective, our open call project was launched independently from UNC leadership’s official planning for the fall semester,^18^ with the goal of providing an alternative, community-driven approach to identifying innovative ideas that could be advocated for and implemented. The open call was designed following standardized approaches to crowdsourcing as developed by the World Health Organization’s Special Programme for Research and Training in Tropical Diseases,^19^ the stages of which are summarized in Table 1. Note that although the collection of contributions from participants is only one point in the crowdsourcing process, our reference to the Carolina Collective crowdsourcing open call refers to these stages depicted here as a whole. This qualitative study used a descriptive approach and is reported according to the Standards for Reporting Qualitative Research (SRQR) reporting guideline.^20^ This study was assessed by the UNC Institutional Review Board and was determined to be exempt from the requirement for approval.

**Table 1.** Overview of the Carolina Collective Open Call Stages, Structure, and Function^a^

Open call stage	Structure	Function
Organize community steering committee, organizing committee, and judging team	Diverse groups of relevant stakeholders, including UNC students, staff, faculty, and alumni	Steering committee: finalize call for submissions, prizes, and rules of the open call; organizing committee: promote the open call and collect submissions; judging team: evaluate submissions
Engage community to contribute	Digital events and social media promotion	Clarify the open call and encourage submissions
Evaluate contributions	Judging team evaluates submissions based on set criteria; steering committee identifies finalists	Narrow the field of submissions and identify excellent ideas
Recognize exceptional finalists	Prize incentives for excellent ideas	Officially acknowledge and celebrate finalist submissions and those who submitted
Share exceptional submissions and identify pathways for potential implementation	Website featuring summaries of finalist and runner-up submissions	Promote exceptional submissions among UNC leadership and assist teams to connect with institutional resources and supports for pursuing their ideas

Abbreviation: UNC, University of North Carolina at Chapel Hill.

^a^ Adapted from World Health Organization, Special Programme for Research and Training in Tropical Diseases, Social Innovation in Health Initiative.^19^

The Carolina Collective included a steering committee, organizing committee, and judging team, each of which included UNC students (graduate and undergraduate), staff (administrative and research), faculty, and alumni. The judging and organizing groups were volunteers who responded to an open call for participants, whereas the steering committee members were selected and invited to participate by the chairs of the organizing committee based on their expertise in public health and/or health equity and how representative of diverse segments of the UNC population they were. The steering committee set the open call parameters, determining that the focus should be on obtaining creative ideas to inform safety in the fall semester. Four prompts were developed to serve as the categories for submitting ideas to the open call (eAppendix 1 in the Supplement).

The organizing committee oversaw the day-to-day activities of the open call, including developing a website that contained information about the open call,^21^ encouraging submissions, answering questions about the open call from potential participants, and collecting submissions from participants via an online submission form. Given COVID-19 safety restrictions on in-person gatherings, all promotion of the open call was conducted online using email, social media platforms, and livestream events. Emails that announced the launch of the open call and contained the link to submit ideas were distributed through UNC department listservs, student organizations, student government, the Faculty Executive Committee, and the Employee Forum. Social media accounts were made for the open call on Facebook,^22^ Twitter,^23^ and Instagram^24^ to announce the launch of the open call, provide information about how to submit ideas, and encourage submissions. As an incentive to encourage contributions, a total of $20 000 in prize money was obtained from the UNC Development Office via the Carolina Fund (a flexible funding resource for meeting immediate campus needs and research opportunities). The organizing committee convened 2 information sessions hosted using videoconferencing to provide potential participants with more details about the open call and answer questions. A livestreamed event was also hosted on social media to promote the open call and encourage submissions. The organizing committee met weekly via videoconference to coordinate promotion activities, and a virtual workplace (slack.com) was used for immediate communication among members and the sharing of resources.

Submissions to the open call were collected from June 16 to July 16, 2020. Participation was open to all UNC students, faculty, staff, alumni, and others (eg, members of the community surrounding the UNC campus). At the submission deadline, all ideas were assessed for initial eligibility (ie, the submission must have contained an idea in response to 1 of the prompts) by 3 members of the organizing committee. Following rigorous standards recommended by the World Health Organization’s Special Programme for Research and Training in Tropical Diseases for evaluating crowdsourced contributions,^19^ eligible submissions were then distributed among the judging team for evaluation of idea quality based on a set of 4 criteria: potential effect on the safety and well-being of the university community; innovation, feasibility, and inclusivity (in terms of gender, race/ethnicity, and disability). Each submission was assigned to 5 judges who independently provided a score for each on a scale of 1 to 10 (1 being lowest quality and 10 being highest quality). Judges could recuse themselves from evaluating submissions when there would be potential for conflict of interest because of work or personal relationships. Scores were then collected from all judges and a mean score calculated for each submission. The steering committee reviewed mean scores and made final decisions about the prize structure for distributing the $20 000 prize money among finalists and runners-up. Finalist teams received a cash prize of $2500 each and were encouraged (though not required) by the Steering Committee to use prizes toward further development and implementation of their ideas. Once the finalists and runners-up were identified by the steering committee, summaries of finalist and runner-up submissions were shared on the open call website (with submitting teams’ permission), with announcements made on all Carolina Collective social media accounts that the open call results were available.

### Data Collection

Submissions to the open call were collected via a submission form hosted on the online survey platform Qualtrics. Participants of the open call could submit ideas in response to 1 or more of 4 submission categories (eAppendix 1 in the Supplement); multiple submissions to the same category were also permitted. Participants could submit ideas as individuals or as a team. Participants were asked to provide brief demographic information for the submitting individual; if submitting an idea as a team, the UNC affiliation of all team members was also collected. Following the judging period, we issued an online survey to all finalists and runners-up with brief questions regarding future implementation of their ideas: (1) Are you and/or your team interested in implementing your idea at UNC during the fall semester? (2) Are you or anyone on your team interested in enrolling in a for-credit elective to facilitate implementation?

### Statistical Analysis

All demographic data collected from open call participants were compiled and analyzed using descriptive statistics. We used a qualitative approach of open coding to analyze emergent themes in the open call submissions.^25^ All submissions were examined, and an initial code book that contained parent and subcodes was developed by 2 coders (C.L. and F.A.-H.) and iteratively refined. The initial set of codes was developed to capture information on the challenges, goals, and solutions of each proposal. Informed by the social ecological model,^26^ we also developed codes to capture the intervention level of each open call submission, including interventions at the individual level (defined as interventions aimed to directly change individual behaviors or perceptions), community level (defined as interventions that either focused beyond the UNC system [eg, the broader township or county surrounding the university campus] or focused on specific groups of individuals [eg, graduate students, minority groups]), and institutional level (defined as interventions that focus on policy implementation or university-mandated requirements). The initial draft of the code book was discussed with the study team and further refined into the final code book. Three coders (C.L., F.A.-H., and T.G.H.) then used this code book to conduct line-by-line coding of all submission text and images using NVivo, version 12 software (QSR International). The final codebook was composed of 10 parent codes, 33 secondary codes, and 22 tertiary codes (eAppendix 2 in the Supplement). From this coding process, a descriptive summary was produced to characterize the content of open call submissions and identify prominent themes that pertained to the challenges identified by participants’ submissions, the goals that their submissions focused on, and the types of solutions that were proposed.

## Results

### Open Call Participation

The open call received 82 submissions in total. After initial screening for eligibility, 80 submissions were forwarded to the judging team for evaluation. Seven submissions received a mean score of 8 (of 10) or greater and were recognized by the steering committee as finalists (ie, top-scoring submissions). In addition, 17 submissions received a good mean score (between 7.0 and 7.9) and were recognized as runners-up (a secondary tier akin to honorable mention). The distribution of all 80 submission scores is indicated in the Figure. Among the 7 finalist submissions, all were submitted by teams of 2 or more participants, as were most (12 [71%]) of the 17 runner-up submissions.

**Figure.**
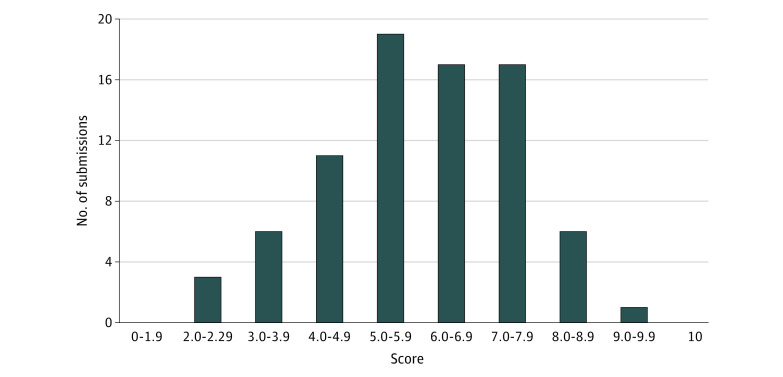
Scores of the Crowdsourcing Submissions

Of the 82 total submissions, 35 (43%) were submitted by a group of 2 or more individuals as a team, and 47 (57%) were submitted by individuals. Of the 35 submissions made by a team, 15 (43%) were submitted by groups of mixed UNC affiliation (eg, students, faculty, and staff working together). In total, 110 individuals participated in the open call, including 63 current UNC students (57%), 22 staff members (20%), 16 alumni (15%), 7 faculty members (6%), and 2 other individuals (2%) (1 former faculty member and 1 community member). Demographic characteristics of submitting individuals are summarized in Table 2 for all 82 submissions.

**Table 2.** Number of Submissions Made by Sociodemographic Characteristics of the Submitting Individuals

Characteristics of submitting individuals	No. (%) of submissions (N = 82)
Age	
<30 y	67 (82)
≥30 y	15 (18)
Self-identified gender	
Woman	55 (67)
Man	20 (24)
Another gender identity (not specified)	3 (4)
Nonbinary	1 (1)
Prefer not to say	3 (4)
Race/ethnicity	
Asian/Pacific Islander	32 (39)
White	28 (34)
Black	4 (5)
Latinx	3 (4)
Middle Eastern	1 (1)
Another race/ethnicity (not specified)	1 (1)
Multiracial/ethnic	8 (10)
Prefer not to say	5 (6)
Disability	
Yes	4 (5)
No	67 (82)
Prefer not to say	11 (13)
UNC affiliation	
Current student	56 (68)
Staff	15 (18)
Faculty	3 (4)
Alumni	6 (7)
Other	2 (2)

Abbreviation: UNC, University of North Carolina at Chapel Hill.

Most of the 82 submissions to the open call were made by current UNC students (56 submissions [68%]) and people younger than 30 years (67 submissions [82%]). Most ideas were also submitted by women (55 submissions [67%]) and individuals identifying as a racial/ethnic minority or as multiracial/ethnic (49 submissions [60%]). Although most submissions were made by individuals who did not identify as having a disability, 4 submissions (5%) were made by persons with a disability.

### Challenges, Goals, and Solutions for a Safe Semester

We qualitatively analyzed the challenges, goals, and solutions proposed by each of the submissions to the open call. Five common themes emerged across challenges and goals pertaining to COVID-19 infection risk: safety, remote learning, mental health, racism/inequality, and transportation. These themes are summarized in Table 3 with examples of proposed solutions.

**Table 3.** Summary of Themes Emerging From Open Call Submissions in Terms of Challenges to Safety and Well-being During COVID-19, Submission Goals, and Examples of Proposed Solutions

Challenges identified in submissions	Submission goals	Examples of proposed solutions
Safety concerns related to the risk of COVID-19 infection	To promote medical or physical health strategies to contain the spread of COVID-19	Disseminating protective gear and/or sanitation supplies (eg, masks, gloves, and hand sanitizer); contact tracing; daily case updates; temperature checks; enforcing safety rules (eg, face covering requirements and social distancing); and changing factors that lead to behavioral change, such as increasing awareness of health risks, changing social norms of face covering, and adding reminders (eg, posters or stickers) of healthy practices to the environment.
Limited student development in the mode of remote learning	To optimize the remote learning experience for student development	Expanding access to remote learning resources, providing virtual mentorship or career development training, using virtual reality- or game-based techniques in online teaching, and organizing virtual student activities or social events.
A lack of mental health support and escalation of COVID-related distress	To provide mental health support	Virtual social events, online support groups, and allowing family members to visit students in a safe space that follows the COVID-19 protection rules (eg, face covering and plastic shield between visitors and students).
The negative impact of racism and inequities on campus and/or in the university system on health and safety during the pandemic	To address health equity across different groups	Programs to ensure equal access to protective gear, offering food stamps or healthy meals to individuals having food insecurity, work safety and pay increase for low-income workers, and raising the awareness of racial/ethnic disparities in health.
Reduced operation of public transportation	To ensure equal access to safe transportation	Increasing affordable on-campus parking, expanding bus services to lower passenger load on a single vehicle, setting up a bus seat sign-up system, and operating direct bus routes between student dorms and grocery stores.

We categorized submissions’ proposed solutions by intervention levels as informed by the social ecologic model,^26^ with some submissions encompassing more than 1 intervention level. The Box describes examples of submissions that pertained to each of the 3 intervention levels: individual, community, and institutional/university level. Of the 82 submissions, 45 submissions (55%) of proposed solutions focused on ideas for changes to be made at the institutional or university level, such as restriction of on-campus dining and studying in the library, contact tracing of individuals entering university buildings by swiping university identification cards, and addressing racial/ethnic equity and inclusivity in the university’s response to COVID-19. Several submissions also focused on physical alterations to campus spaces, such as touch-free door openers, hand washing stations, and outdoor workspaces. Thirty-two interventions (39%) focused on the community level. These interventions included creating virtual support groups and study rooms for specific populations (eg, online graduate student support networks), public virtual reality tours of campus, and improving access to safe and equitable transit options. Finally, 14 submissions (17%) were aimed at individual-level changes. These submissions included ideas for increasing the adoption of protective gear (eg, masks and sanitation) (Box) as well as creative strategies for encouraging individuals to adhere to safety standards (eg, university-themed mask designs). Individual-level interventions also included communication-based strategies designed to increase individuals’ knowledge and awareness of COVID-19 safety, such as public safety apps, informational posters, and health awareness campaigns.

Box. **Examples of Open Call Submissions Across the Individual, Community, and Institutional Intervention Levels**

**Individual Level**

- Creative design of face covering (eg, mood masks)
- Increase individual knowledge, awareness, and motivations related to COVID prevention (eg, posters in public places)
- COVID data sharing and contact tracing apps (eg, NOVID)

**Community Level**

- Virtual support groups or forums
- Virtual social events
- Storytelling of UNC members in the pandemic
- Virtual campus tour for the public
- Public transit (eg, adapting bus routes to meet residents’ essential needs)

**Institutional Level**

- Mandatory COVID-safe training, face covering, temperature check, physical distancing, and distribution of protective gear or sanitizing products
- Adapted dining services
- Changes in physical space (eg, seat masking and 1-way traffic signs)
- Teaching and student support (eg, outdoor classrooms and online teaching and mentoring programs)

Abbreviation: UNC, University of North Carolina at Chapel Hill.

### Implementation of Open Call Submissions

After the judging period, 18 finalist and runner-up teams agreed to be showcased on the Carolina Collective website. Among these 18 teams, 17 indicated interest in recruiting community members (UNC students, faculty, and staff) to join their project and requested that this be included in their feature (along with team contact information). All 24 finalist and runner-up teams responded that they were interested in implementing their idea at UNC; all but 3 teams indicated that they would be interested in an elective credit option (although of the 3 declining teams, 2 were composed of staff-only participants and 1 was composed of graduate students for whom elective credit would not be relevant). All of the finalists and runners-up received support from Innovate Carolina, a UNC office focused on innovation and entrepreneurship.^27^ For runners-up, this support included outreach from Innovate Carolina to provide relevant resources for further development of their ideas (eg, linkage to entrepreneurship programs and events). For finalists, Innovate Carolina organized one-on-one meetings to discuss the next steps in implementing their ideas and ways that the office could provide support. In addition, the Carolina Collective organizing committee met with 4 university leaders to share top-scoring ideas and promote implementation and shared communications-related submissions with a pan-university communications committee.

## Discussion

In this qualitative study, the open call fostered community engagement at a large public university, providing a bottom-up approach to identifying promising ideas for reimagining university life during the pandemic. Although crowdsourcing has been used by other universities to help create a safer fall 2020 semester,^14,15,16,17^ these open calls have not been formally assessed. This study expands this literature by evaluating open call participation and examining the themes that emerged from submissions. That multiple segments of the UNC community participated in the open call exemplifies the significant advantage of this approach as a strategy for involving a broad range of stakeholders in planning and creative problem solving,^5^ providing a participatory alternative to top-down strategic planning processes. In addition, the ongoing implementation efforts of open call finalists and runners-up exemplifies the longer-term engagement effects of an open call, in contrast to point-in-time engagement strategies, such as university surveys.

The findings of this study suggest that open calls may be particularly useful for community engagement during COVID-19 and other situations that call for rapid, digital community engagement during an emergency response. First, the large number of submissions to our open call demonstrates that this approach is a feasible method. This finding is consistent with the German COVID-19 technology hackathon #WeVsVirus, a government-hosted digital crowdsourcing event that successfully used online-only methods of organization, promotion, and participation.^28^ The similar reliance on digital methods for launching the open call was more feasible compared with in-person activities that were delayed or cancelled in the period leading up to the fall semester, enabling participation in the open call during the most stringent lockdown period. As the pandemic continues, using digital methods for open calls on other research topics in need of community input (eg, methods to increase vaccine uptake) may be similarly effective because of increasing familiarity with virtual events and digital engagement platforms among members of the public.

Second, the open call resulted in strong engagement among racial/ethnic minorities, who contributed 60% of all submissions, including strong participation from minority students (37 of 56 student submissions [66%]) and staff (8 of 15 staff submissions [53%]). Racial/ethnic minorities were represented on the steering committee, organizing committee, and judging team. These findings are consistent with evidence that suggests that open calls are an effective strategy to engage marginalized populations.^13^ Addressing racial/ethnic inequality also emerged as a significant theme in our analysis of intervention goals. Given that COVID-19 disproportionately impacts racial/ethnic minorities,^29^ open calls may be a valuable approach for enhancing community engagement and coproduction of ideas for responding to the pandemic from minority populations.

Third, open call data collected in this study may provide a formal mechanism to better understand community concerns during COVID-19. This finding is consistent with a study on crowdsourced narratives providing an opportunity for social listening.^30^ The findings of the current study provided an early warning signal about faculty, staff, and student concerns related to reopening. These findings demonstrate how crowdsourcing can provide novel insights into community concerns that thus far have been absent from institutional responses to the pandemic.

Fourth, this study suggests how crowdsourcing open calls can facilitate community-engaged collaboration to address complex medical and public health issues that require a combination of biomedical and behavioral interventions. Although we did not collect detailed information about disciplinary background, several teams were composed of mixed university affiliation (groups of students, staff, and faculty working together) and included expertise from multiple departments within the university. This mixed affiliation may have helped to promote multidisciplinary collaboration in response to COVID-19 and is consistent with broader literature demonstrating that other forms of health crowdsourcing (eg, hackathons) at universities can foster innovation through teamwork and multidisciplinary collaboration.^31,32^ Crowdsourcing open calls may help to spur collaboration within and between medical schools, schools of public health, and related allied health professions.

### Limitations

There are several limitations to this study. First, data on the implementation or effectiveness of the proposed interventions collected from the open call are not yet available. Second, although the open call received submissions that expressed concern with in-person reopening that were communicated to university leadership, this communication did not prevent a reopening process characterized by substantial COVID-19 transmission, ultimately necessitating a shift to remote learning.^33^ Notably, the open call was conducted independently of university leadership’s decision-making regarding the fall 2020 semester. To fully benefit from the creative contributions of this engagement approach, future open calls on the COVID-19 pandemic could be formally incorporated into university planning processes. However, the submissions collected from this open call may be useful for informing university activities during the ongoing pandemic. Third, which of these ideas would have been developed without the support of the open call is not known.

## Conclusions

These findings suggest that open calls are a feasible strategy for engaging a university community during the COVID-19 pandemic. Using entirely digital organization, promotion, and participation strategies, this open call facilitated widespread participation among diverse university-related stakeholders. Submissions to the open call provided novel, innovative ideas for addressing university community members’ concerns about reopening. Research is needed to identify which of the submitted interventions would be most acceptable and effective to implement.

## Notes

Supplement. **eAppendix 1.** Open Call Prompts **eAppendix 2.** Code Book Developed for Coding of Submissions to the Carolina Collective Open Call
