# Supplementary material for: Assessment of a Crowdsourcing Open Call for Approaches to University Community Engagement and Strategic Planning During COVID-19
Source: JAMA Netw Open. 2021 May 14;4(5):e2110090. doi: 10.1001/jamanetworkopen.2021.10090 (PMC8122225; doi:10.1001/jamanetworkopen.2021.10090)
Supplement: Supplement. — eAppendix 1. Open Call Prompts eAppendix 2. Code Book Developed for Coding of Submissions to the Carolina Collective Open Call [file jamanetwopen-e2110090-s001.pdf]

Supplementary Online Content

Day S, Li C, Hlatshwako TG, et al. Assessment of a crowdsourcing open call for approaches to university community engagement and strategic planning during COVID-19. *JAMA Netw Open*. 2021;4(5):e2110090. doi:10.1001/jamanetworkopen.2021.10090

eAppendix 1. Open Call Prompts

eAppendix 2. Code Book Developed for Coding of Submissions to the Carolina Collective Open Call

This supplementary material has been provided by the authors to give readers additional information about their work.

© 2021 Day S et al. *JAMA Network Open.*

eAppendix 1. Open Call Prompts

The following prompts were used to solicit responses to the open call. Participants could submit ideas in response to one or more of the following idea categories:

1. *Public health campaign:* How might we make the (virtual and physical) campus safe while continuing the things you are passionate about? Submission format: text (1-page PDF), images (2 MB), and/or video (3 minutes).

2. *Inclusive digital dialogue:* How might we create a more effective bidirectional digital dialogue within and between our university groups? Submission format: text (2-page PDF).

3. *Collective action:* How might we draw on our collective strengths to translate voices into action? Submission format: text (2-page PDF), images (2 MB), and/or video (3 minutes).

4. *Re-thinking safety:* What are ways of creating and maintaining safety that are respectful and anti-racist? Submission format: text (1-page PDF).

© 2021 Day S et al. *JAMA Network Open.*

eAppendix 2. Code Book Developed for Coding of Submissions to the Carolina Collective Open Call

Code Name	Number of submissions coded under this code
1. Development stage	0
1.1 Co-creation	4
1.2 Conceptual idea	76
1.3 Development-other	4
1.4 Pilot test	3
1.5 Preparation	0
1.5.1 Evidence-based	4
1.5.2 Formative work	5
1.6 Prototype ready	10
2. Focus population	3
2.1 Faculty	20
2.2 Population-other	9
2.3 Staff	21
2.4 Students	59
3. Ideal quotes	0
4. Interesting	2
5. Intervention level	0
5.1 Community level	32
5.2 Individual level	14
5.3 Institutional level	45
5.4 Level-other	0
5.5 Physical environment	5
5.5.1 Accessories	3
5.5.2 Physical space	11
5.6 Social environment	13
6. Intervention objectives	1
6.1 Academic performance	12
6.2 Behavioral path	1
6.2.1 Behavioral skills	6
6.2.2 IMB-extend	1
6.2.3 Information	12
6.2.4 Motivation	13
6.3 COVID-specific	6
6.3.1 Contact tracing	7
6.3.2 COVID test	5
6.3.3 Movement tracking	1
6.3.4 Physical distancing	13
6.3.5 Social distancing	8
6.3.6 Temperature check	4
6.4 Equity	20
6.5 Health outcomes	2

© 2021 Day S et al. *JAMA Network Open.*

6.5.1 Mental health	19
6.5.2 Physical health	16
6.5.3 Public health	14
6.6 Objective-other	3
6.7 Student life - socializing	9
6.8 Transportation	5
7. Intervention strategies	1
7.1 Communication	16
7.2 Cues or reminders	9
7.3 Digital solution	39
7.4 Physical alternation	4
7.4.1 Accessories	3
7.4.2 Intra-personal interaction	0
7.4.3 Physical space	4
7.5 Policy or Procedure or Requirement	3
7.6 Restriction of activities	4
7.7 Social support	13
7.8 Strategy-other	3
7.9 Supplies or Tools	1
7.9.1 Protective gear and sanitation	10
7.9.2 Tool-other	1
8. Other	1
9. Problem statement	31
10. Resources	20

Note that where a parent code contains a count of 0 coded submissions, this indicates that all submissions under this parent code were subsequently re-coded with a secondary (or tertiary) code.

© 2021 Day S et al. *JAMA Network Open.*
